# Supplementary figures and images for: Weissella cibaria suppresses colitis-associated colorectal cancer by modulating the gut microbiota-bile acid-FXR axis
Source: mSystems. 2025 Jul 3;10(7):e00288-25. doi: 10.1128/msystems.00288-25 (PMC12282153; doi:10.1128/msystems.00288-25)

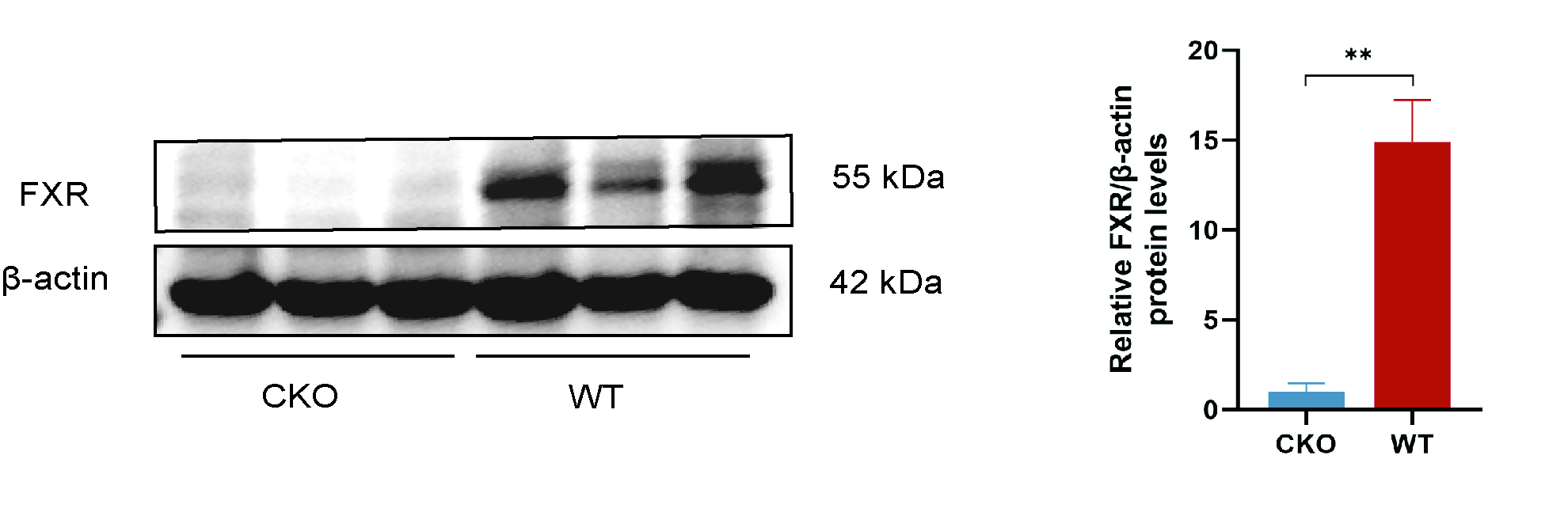

Supplement: Fig. S1 — Protein expression of FXR in colon tissues. [file msystems.00288-25-s0001.tif]

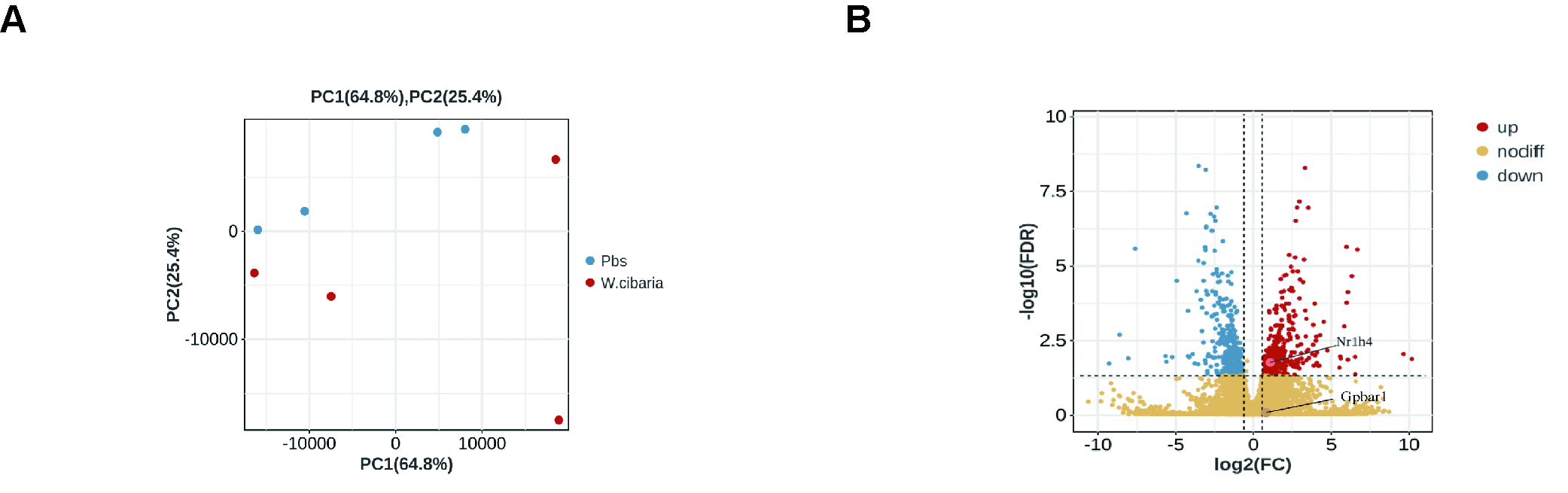

Supplement: Fig. S2 — PCA analysis for RNA-seq detection of colon tumor and differential gene volcano map. [file msystems.00288-25-s0002.tif]

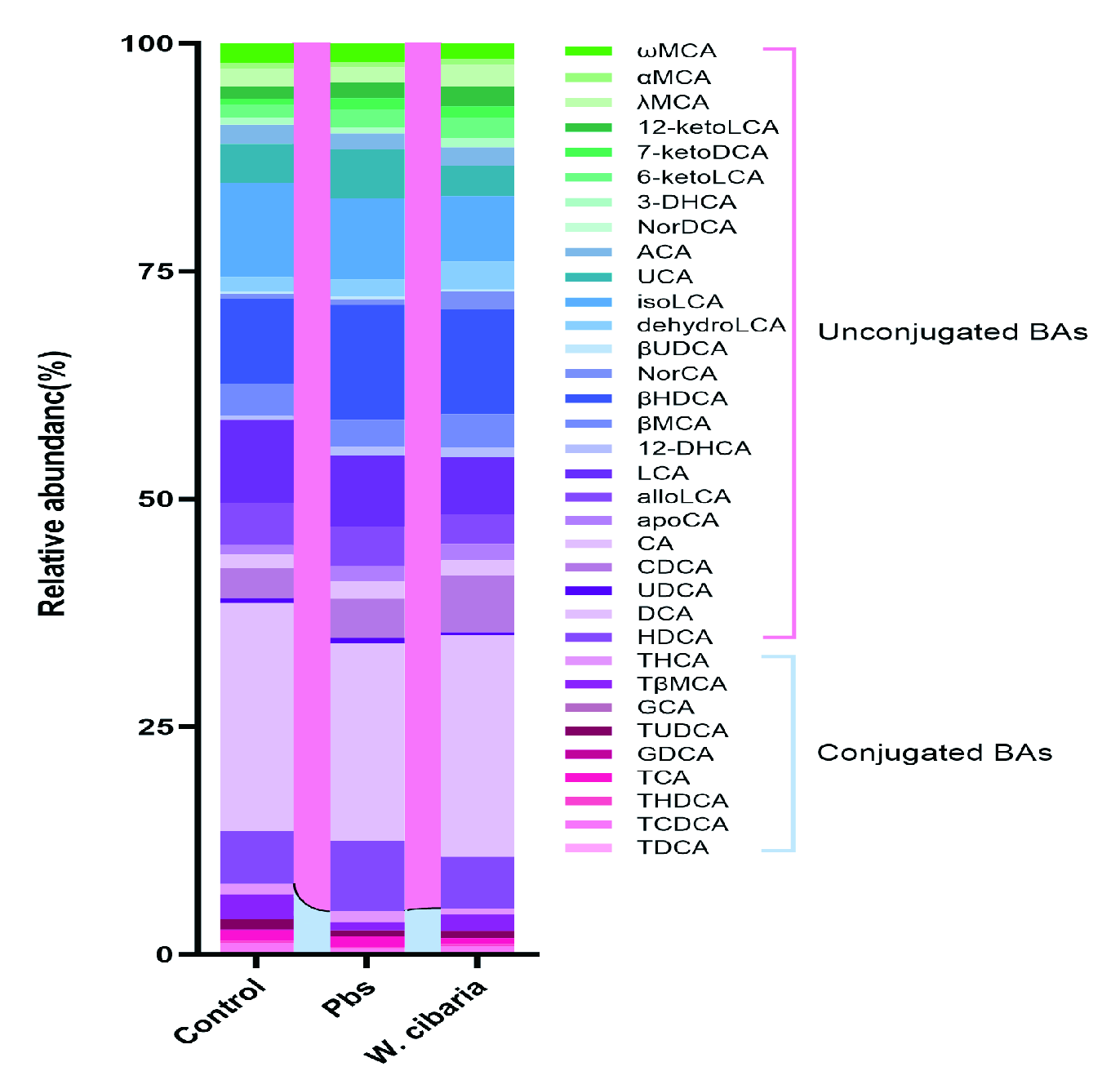

Supplement: Fig. S3 — Relative abundance ratios of various types of bile acids. [file msystems.00288-25-s0003.tif]

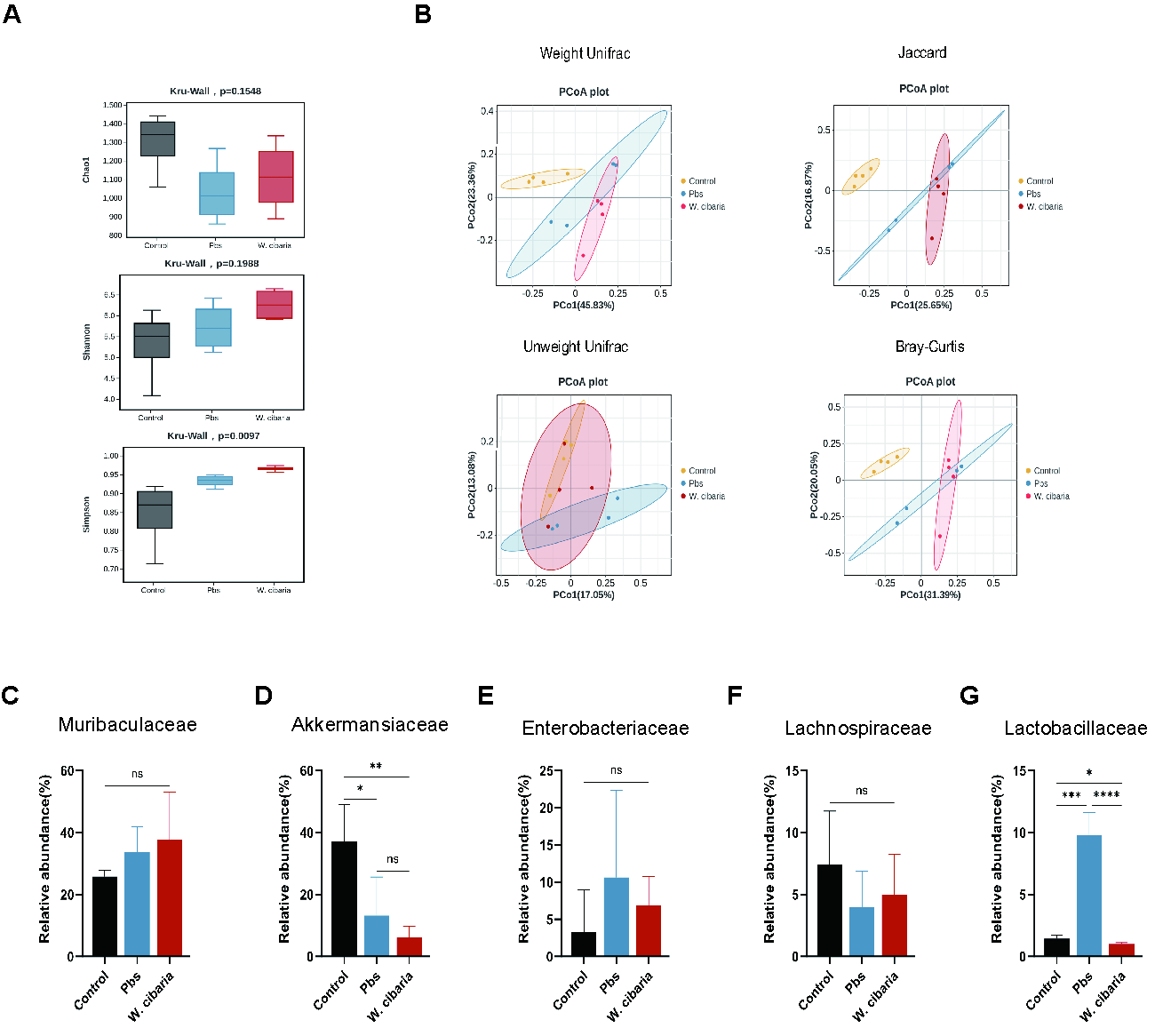

Supplement: Fig. S4 — α-Diversity indexes, PCoA, and comparative abundance of the top 5 most abundant taxa at the family level. [file msystems.00288-25-s0004.tif]
