# Supplementary material for: Weissella cibaria suppresses colitis-associated colorectal cancer by modulating the gut microbiota-bile acid-FXR axis
Source: mSystems. 2025 Jul 3;10(7):e00288-25. doi: 10.1128/msystems.00288-25 (PMC12282153; doi:10.1128/msystems.00288-25)
Supplement: Legends — for Figures S1 to S4. [file msystems.00288-25-s0005.docx]

**List of Supporting Information:**

Figures:

Fig. S1. The protein expression of FXR in colon tissues.

Fig. S2. (A) PCA analysis for RNA-seq detection of colon tumor. (B) Differential gene volcano map.

Fig. S3. Relative abundance ratios of various types of bile acids.

Fig. S4. (A) α-diversity indexes include Chao1, Shannon and Simpson indices. (B) The PCoA based on the weight unifrac, jaccard, unweight unifrac and bray–curtis distances (β-diversity indexes). (C-G) Comparative abundance of the top 5 most abundant taxa at the family level.
