# Supplementary material for: Weissella cibaria suppresses colitis-associated colorectal cancer by modulating the gut microbiota-bile acid-FXR axis
Source: mSystems. 2025 Jul 3;10(7):e00288-25. doi: 10.1128/msystems.00288-25 (PMC12282153; doi:10.1128/msystems.00288-25)
Supplement: Table S1 — Primers for mouse identification. [file msystems.00288-25-s0006.pdf]

Table S1. Primers for mouse identification

Primer name	Sequence
Fxr-5’arm-F1	GGCTATTAGAGTTTTAAGTGCTGGAGG
Fxr-5’arm-R1	CATGTCAGGTCTAATACTGAAATGGAC
Fxr-3’arm-F1	TCTGAGACGGGATCACAATGTG
Fxr-3’arm-R1	GCTGTTCCCTCACTCTGTCTTTAG
H11-Vil1-iCre-5’arm-F1	GGCCTCCAAGTCTTGACAGTAGAT
H11-Vil1-iCre-5’arm-R1	ATCTCCCTCAGTCTCCAGTGTGCAA
H11-Vil1-iCre-wt-F1	GGGCAGTCTGGTACTTCCAAGCT
H11-Vil1-iCre-wt-R1	ATATCCCCTTGTTCCCTTTCTGC
