# Supplementary material for: Weissella cibaria suppresses colitis-associated colorectal cancer by modulating the gut microbiota-bile acid-FXR axis
Source: mSystems. 2025 Jul 3;10(7):e00288-25. doi: 10.1128/msystems.00288-25 (PMC12282153; doi:10.1128/msystems.00288-25)
Supplement: Table S2 — Species-specific primers and probes used for qPCR. [file msystems.00288-25-s0007.pdf]

Table S2. Species-specific primers and probes used for qPCR

Primer/Probe
W. cibaira
forward, 5’-GTGAAAGCCCTCAGCTCAAC-3’
reverse, 5’-CTACGCATTTCACCGCTACA-3’
5’-FAM-TGGAAACTGGATGACTTGAGTGCA-BHQ-3′
