# Supplementary material for: Weissella cibaria suppresses colitis-associated colorectal cancer by modulating the gut microbiota-bile acid-FXR axis
Source: mSystems. 2025 Jul 3;10(7):e00288-25. doi: 10.1128/msystems.00288-25 (PMC12282153; doi:10.1128/msystems.00288-25)
Supplement: Table S3 — Full names of the bile acids in Fig. 6. [file msystems.00288-25-s0008.pdf]

Table S3. Full names of the bile acids in Fig. 6.

Abbreviation	Full name
TDCA	Taurodeoxycholic acid
TCDCA	Taurochenodeoxycholic acid
THDCA	Taurohyodeoxycholic acid
TCA	Taurocholic acid
GDCA	Glycodeoxycholic acid
TUDCA	Tauroursodeoxycholic acid
GCA	Glycocholic acid
TβMCA	Tauro-β-muricholic acid
THCA	Taurohyocholie acid
HDCA	Hyodeoxycholic acid
DCA	Deoxycholic acid
UDCA	Ursodeoxycholic acid
CDCA	Chenodeoxycholic acid
CA	Cholic acid
apoCA	Apocholic acid
alloLCA	Allolithocholic acid
LCA	Lithocholic acid
12-DHCA	12-Dehydrocholic acid
βMCA	β-Muricholic acid
βHDCA	β-Hyodeoxycholic acid
NorCA	Norcholic acid
βUDCA	β-Ursodeoxycholic acid
dehydroLCA	Dehydrolithocholic acid
isoLCA	Isolithocholic acid
UCA	Ursocholic acid
ACA	Allocholic acid
NorDCA	Nordeoxycholic acid
3-DHCA	3-Dehydrocholic acid
6-ketoLCA	6-ketolithocholic acid
7-ketoDCA	7-ketodeoxycholic acid
12-ketoLCA	12-ketolithocholic acid
λMCA	λ-muriclolic acid
αMCA	α-muriclolic acid
ωMCA	ω-muriclolic acid
