# Supplementary material for: Weissella cibaria suppresses colitis-associated colorectal cancer by modulating the gut microbiota-bile acid-FXR axis
Source: mSystems. 2025 Jul 3;10(7):e00288-25. doi: 10.1128/msystems.00288-25 (PMC12282153; doi:10.1128/msystems.00288-25)
Supplement: Table S4 — Primers for real-time PCR. [file msystems.00288-25-s0009.pdf]

Table S4. Primers for real-time PCR

| Primer name | Forward Primer（5'-3'） | Reverse Primer（5'-3'） |
|---|---|---|
| β-actin (mouse) | GCTGTGCTATGTTGCTCTAG | GCTCGTTGCCAATAGTGATG |
| Osta (mouse) | GTGTCTACCCTTCTGGCCCT | CGGTCAGGATGACAAGCACC |
| Ostb (mouse) | GAGAAAGCTGCAGCCAATGC | GTCATGACCACCAGGACTGC |
| Asbt (mouse) | GTGCCGAACAGTAGCCTTGG | GCTGCAAAGACGAGCTGGAA |
| ZO-1 (mouse) | GTTGGTACGGTGCCCTGAAAGA | GCTGACAGGTAGGACAGACGAT |
| occludin (mouse) | TGGCAAGCGATCATACCCAGAG | CTGCCTGAAGTCATCCACACTC |
| Claudin-1 (mouse) | GGACTGTGGATGTCCTGCGTTT | GCCAATTACCATCAAGGCTCGG |
| FXR (mouse) | GCTTGATGTGCTACAAAAGCTG | CGTGGTGATGGTTGAATGTCC |
| FGF15 (mouse) | GTCGCTCTGAAGACGATTGCCA | CAGTCTTCCTCCGAGTAGCGAA |
| SHP (mouse) | AGATCCTGCTAGAGGAAGCCA | CCTGGCACATCTGGGTTGAA |
| TGR5 (mouse) | CACTGCTCTTCTTGCTGTGTTGG | GAGCGATAACAGAGTTCCAGGC |
| β-actin (human) | CACCATTGGCAATGAGCGGTTC | AGGTCTTTGCGGATGTCCACGT |
| FXR (human) | ACTTCCGTCTGGGCATTCTGAC | GCTGTAAGCAGAGCATACTCCTC |

Abbreviations: organic solute transporter alpha (Osta), organic solute transporter beta (Ostb), apical sodium-dependent bile acid transporter (Abst), zonula occludens-1 (ZO-1), farnesoid X receptor (FXR), fibroblast growth factor 15 (FGF15), small heterodimer partner (SHP), takeda G protein-coupled receptor 5(TGR5).

.
